# Children’s Food

**Published:** 1872-01

**Authors:** 


### CHILDREN'S FOOD.

BY UNCLE BOB.

It is a very difficult matter to determine the origin of likes and dislikes. Why one person should have a fondness for an article of food and a second person be indifferent to its peculiar taste, and a third actually crave it, is a mystery. We are all created as peculiar in this respect as we are made differing in temperaments.

Still,in face of these born peculiarities I make the assertion that the development of *tastes* is a matter of education in childhood.

Some one has written "that it is utterly wrong to compel a child to eat any article of food that is put aside by it," urging as a reason "that nature has placed the fancy and no compulsion is to be used to create an artificial appetite or taste." He says "let the natural tastes be left unmeddled with—nature is the judge." This I do not believe. One might as well urge that a child should be the judge as to whether it wished its face washed, and if it preferred dirt to cleanliness, the disposition ought to be followed.

Common sense will always be ready to assist nature in matters of habit. Some children are born with a naturally slow temperament, and if left untutored, will become known for the peculiarity of being behindhand. Such a child needs direction in order to become efficient in life. Another child is the reverse of this and needs "checking in," or it will develop as great faults in a contrary direction.

*What* the child is taught to eat is as essential as the *how*, for good breeding is manifest in both these points. Naturally a child will select for food that which is sweet and pleasing to the taste, merely. Therefore it must *learn* to eat substantial food which is less pleasing but more beneficial. A constant succession of the same dish will sooner become tiresome to a child than to an adult, consequently it turns from it in the exercise of natural born rights. To urge this then, becomes cruelty. Better nothing to eat than to cloy the early appetite.

From the time when the infant's first food is dropped, his education as what he shall eat begins. He commences to "learn to eat" we say. At this time every judicious parent should select good substantial diet for his children and have it in a pleasing variety. When it is placed upon the table he should exercise reasonable judgment as to *how* it is eaten. Many children are allowed to refuse good and wholesome food from mere whims. Few have *positive* dislikes at the first, to the majority of usual dishes, and need not grow up with confirmed and disagreeable prejudices. As a child grows older there is ample opportunity for knowing why it refuses any kind of food and consequently strong dislikes ought not to be developed.

Much could be said upon *whims*. They are not to be overlooked, but are to be quieted by reason. Little folks are imitators of those who are about them. If they see that there is a regularity in the method of furnishing the food to the table, they learn to consider that everything is wholesome. Often the simple fact of the name of a dish becomes the point upon which a childish whim is turned. Watch them for a reason, if there is one, why your child does not wish for some dish. Let it be made a point to overcome this dislike by every expedient which your common sense can suggest. It may be necessary to deny every other article upon the table, for hunger will bring an appetite perhaps, which will make a previously unpalatable dish to be welcomed.

I am not unmindful of the arguments which can be brought to bear upon this subject of "food." So long as children are born and parents are heedless, the matter lies open for debate.

We are all sensitive upon these habits and prejudices. We do not like to admit that we are at all in the wrong. Those who have fewest dislikes will naturally fall to one side of the argument and will meet a formidable array of talent upon the opposite. Still this should not prevent careful thought by every one. It comes home to us all continually. We who provide are taxed to make up a bill of fare which shall be acceptable to those who are to eat, and we who eat are taxed to exhibit a pleasing appreciation of a dish which we cannot refuse and yet cannot eat and be happy.

Truly no one possesses all of wisdom, therefore no one can decide the subject of tastes. However, we can render service by endeavoring to educate our children to eat what is set before them and ask no questions for conscience sake. For this we have the scripture authority, and what is better?

